# TRIM52 Protects Against Doxorubicin‐Induced Cardiac Inflammation, Oxidative Stress and Cardiac Injury

**DOI:** 10.1111/jcmm.71016

**Published:** 2026-01-09

**Authors:** Zhaoxia Zhang, Hongzhen Chen, Yingchu Hu, Jiedong Zhou, Yiqi Lu, Tingsha Du, Zhenyu Jia, Jia Su, Weiping Du

**Affiliations:** ^1^ Department of Cardiology The First Affiliated Hospital of Ningbo University, The Key Laboratory of Precision Medicine for Atherosclerotic Diseases of Zhejiang Province Ningbo China; ^2^ Health Science Center Ningbo University Ningbo China

**Keywords:** cardiotoxicity, doxorubicin, inflammation, oxidative stress, TRIM52

## Abstract

Tripartite motif 52 (TRIM52) has been identified as a key regulator of inflammatory responses. However, its involvement in doxorubicin (DOX)‐induced cardiotoxicity (DIC) and the underlying molecular mechanisms remain poorly understood. To investigate the functional role of TRIM52, we employed an adeno‐associated virus serotype 9 (AAV9) delivery system to achieve cardiac‐specific Trim52 knockout via tail‐vein injection. C57BL/6 mice received intraperitoneal DOX (5 mg/kg, administered once a week, with a total cumulative dose of 15 mg/kg). Myocardial injury was evaluated by histopathological assessment and molecular profiling of cardiac tissues, complemented by in vitro mechanistic studies using neonatal mouse cardiomyocytes. In vivo and in vitro studies revealed that DOX treatment significantly upregulated TRIM52 expression. Trim52 deficiency effectively mitigated DOX‐induced cardiac injury and dysfunction, concomitantly attenuating oxidative stress and inflammatory responses. Mechanistically, Trim52 deletion markedly enhanced PI3K and AKT phosphorylation, indicating that PI3K/AKT pathway activation underlies the cardioprotective effects of TRIM52 deficiency. Our findings demonstrate that TRIM52 deletion activates PI3K/AKT signalling and attenuates DOX‐induced oxidative and inflammatory myocardial damage. These data identify TRIM52 as a potential therapeutic target for mitigating DIC.

## 1 Introduction

Doxorubicin (DOX) is one of the most widely used anthracyclines in clinical oncology, particularly for the treatment of lymphoma, sarcoma, and breast cancer. Nevertheless, up to one quarter of recipients develop clinically significant DOX‐induced cardiotoxicity (DIC), which limits its therapeutic utility [1]. Moreover, approximately 30% of patients show evidence of cardiac injury that can progress to dilated cardiomyopathy and congestive heart failure, and may be fatal in severe cases [2]. Elucidating the mechanisms underlying DOX‐induced myocardial injury is therefore essential for preventing and managing this complication.

DIC through multiple mechanisms, with oxidative stress and inflammation recognised as dominant contributors [3, 4, 5]. Our previous work similarly demonstrated that dampening oxidative stress and inflammatory signalling alleviates DOX‐induced cardiac damage [6, 7]. Accumulating evidence indicates that the PI3K/AKT signalling axis governs DOX‐triggered oxidative and inflammatory responses: DOX suppresses PI3K/AKT activation, whereas reactivating this cascade mitigates myocardial oxidative stress and inflammation [8, 9]. Therefore, identifying upstream regulators of PI3K/AKT may reveal novel therapeutic targets for DIC.

Tripartite motif (TRIM) family proteins participate in diverse cellular processes, including proliferation, differentiation, tumorigenesis, inflammation, and apoptosis [10]. Several TRIM members have been implicated in cardiovascular disorders such as cardiac hypertrophy, ischemia–reperfusion injury, and heart failure [11]. TRIM52, a newly characterised family member, has attracted interest for its role in regulating inflammation, and emerging evidence links TRIM52 to inflammatory bowel disease, cancer, and sepsis. In parallel, several TRIM proteins have been shown to modulate PI3K/AKT signalling [12, 13, 14]. Given that DIC is strongly driven by oxidative stress and sterile inflammation, and that current preventive strategies (including dose limitation, dexrazoxane, and conventional cardioprotective agents) remain suboptimal, identifying upstream modulators of these processes is critical. However, whether TRIM52 contributes to DOX‐induced myocardial oxidative stress and inflammation, particularly via PI3K/AKT signalling, remains unclear. Therefore, the present study aims to clarify the role of TRIM52 in DOX‐induced myocardial injury and delineate its underlying mechanisms.

## 2 Methods

### 2.1 Animals and Treatment

Male C57BL/6 mice (8–10 weeks old) were obtained from the Chinese Academy of Medical Sciences. All experimental procedures conformed to the National Institutes of Health Guidelines for the Care and Use of Laboratory Animals and were approved by the Animal Care and Use Committee of the First Affiliated Hospital of Ningbo University. After 1 week of acclimation in a controlled environment, mice were housed individually in bedding‐lined plastic cages with ad libitum access to food and water under a 12‐h light/dark cycle at 22°C. TRIM52 knockdown was achieved using adeno‐associated virus serotype 9 (AAV9) vectors encoding TRIM52‐specific short hairpin RNAs (AAV9‐shTRIM52; GeneChem, Shanghai, China), with a scrambled shRNA (AAV9‐shRNA) serving as the control. Four weeks before doxorubicin (DOX) treatment, mice received tail‐vein injections of AAV9‐shRNA or AAV9‐shTRIM52 (1 × 10^10^ viral genomes per mouse). The efficiency of TRIM52 knockdown is shown in Figure S1. To model chronic DOX cardiotoxicity, mice were administered DOX intraperitoneally at 5 mg/kg once weekly for 3 consecutive weeks (cumulative dose, 15 mg/kg) [15]. After the final DOX injection, mice underwent weekly physiological monitoring and body weight measurements before heart tissue collection for subsequent analyses. Animals were randomised into four groups: CTL + AAV9‐shRNA, CTL + AAV9‐shTRIM52, DOX + AAV9‐shRNA, and DOX + AAV9‐shTRIM52.

### 2.2 Echocardiography

Echocardiography was performed using an ESAOTE Mylab30CV ultrasound system equipped with a 15‐MHz transducer, as previously described. Mice were maintained under continuous anaesthesia with 1.5%–2.0% isoflurane during imaging. Key indices of left ventricular systolic function, including left ventricular ejection fraction (LVEF) and fractional shortening (LVFS), were quantified in a standardised manner.

### 2.3 Immunofluorescence Staining

Paraffin‐embedded left ventricular sections were subjected to immunofluorescence staining with an anti‐CD68 antibody (macrophage marker; Bioss, China) to assess inflammatory cell infiltration. Fluorescence images were acquired using an Olympus DX51 microscope (Tokyo, Japan) under standardised settings. For each sample, 20 randomly selected, nonoverlapping fields were analysed at ×200 magnification. Macrophage infiltration was quantified using ImageJ, and all raw images were archived in high‐resolution formats to support reproducibility and verification.

### 2.4 Oxidative Stress Detection

Reactive oxygen species (ROS) were assessed by dihydroethidium (DHE) fluorescence staining. Fresh‐frozen left ventricular cryosections were incubated with 10 μM DHE (Sigma‐Aldrich) at 37°C for 30 min and imaged using an Olympus DX51 fluorescence microscope (Tokyo, Japan) at ×200 magnification. For each sample, 20 randomly selected fields were analysed. Fluorescence intensity was quantified with ImageJ (NIH). For complementary biochemical analyses, snap‐frozen cardiac tissues were homogenised in ice‐cold RIPA buffer and centrifuged at 3000 *g* for 15 min at 4°C. The supernatants were collected for oxidative stress profiling. Commercial ELISA kits (Abcam) were used to quantify 4‐hydroxynonenal (4‐HNE) adducts, superoxide dismutase (SOD) activity, and malondialdehyde (MDA) levels according to the manufacturers' instructions.

### 2.5 Biochemical Testing

Serum biomarkers of cardiac injury, including cardiac troponin T (cTnT), lactate dehydrogenase (LDH), and creatine kinase‐MB (CK‐MB), were measured using standardised commercial assay systems. Inflammatory mediators, including interleukin‐1β (IL‐1β), interleukin‐6 (IL‐6), and tumour necrosis factor‐α (TNF‐α), were quantified using sandwich ELISA kits (Quantikine; NeoBioscience Technology Co., China) according to the manufacturers' instructions.

### 2.6 Cell Culture and Treatment

Neonatal mouse cardiomyocytes (NMCMs) were isolated using an optimised enzymatic digestion protocol. Hearts from 1‐day‐old mice were minced into small fragments and digested with Collagenase I (Worthington) at 37°C for 3 min. The cell suspension was then pre‐plated in DMEM containing 20% FBS at 37°C with 5% CO₂ for 90 min to remove fibroblasts by preferential adhesion. After a 24‐h synchronisation period, NMCMs were treated with doxorubicin (DOX, 1 μM) for 24 h. For gene silencing of TRIM52, cells were transfected for 4 h with 50 nmol/L siRNA (scramble, siTRIM52) using Lipo6000 (RiboBio, Guangzhou), then returned to standard medium for 24 h before subsequent manipulations.

### 2.7 TdT‐Mediated dUTP Nick End Labelling (TUNEL) Staining

The rate of cell death in cardiomyocytes was assessed using TUNEL staining. For the in vitro process, the cells were first fixed using a 4% paraformaldehyde solution. Subsequently, cell membranes underwent permeabilization with 1% triton X‐100, followed by incubation with 100 nM glycine. The incubation buffer, prepared as per the guidelines provided in the TUNEL kit (Cat. 12156792910 from Roche Diagnostics, Germany), was then utilised in a dark environment within a constant temperature incubator set at 37°C. To visualise the nuclei, DAPI staining was performed at a concentration of 0.5 μg/mL. Finally, a confocal microscope (LSM900 model) was used to analyse and capture images of the cell slices.

### 2.8 Western Blotting Assay

Total protein lysates were extracted from cardiac tissues or cultured cells. Aliquots (25–50 μg) were resolved by SDS‐PAGE and transferred to nitrocellulose membranes. Membranes were blocked with 5% BSA, then incubated overnight at 4°C with primary antibodies: TRIM52 (Santa, #sc‐135589; 1:500), p‐PI3K (Abcam, #ab182651; 1:500), PI3K (CST, #4257; 1:2000), p‐Akt (CST, #4060; 1:1000), Akt (CST, #9272; 1:2000), and GAPDH (Abcam, #Ab181602; 1:10,000). After TBST washes, species‐matched HRP‐conjugated secondary antibodies were applied (1 h, room temperature). Protein bands were visualised using Bio‐Rad ECL substrate and quantified densitometrically with Image Lab software (Bio‐Rad).

### 2.9 Statistical Analysis

All experimental data are presented as mean ± SEM. Statistical analyses were conducted with GraphPad Prism software, employing one‐way ANOVA for multigroup comparisons and unpaired Student's *t*‐tests for pairwise group evaluations. Survival curves were analysed via Kaplan–Meier methodology with log‐rank testing for inter‐curve comparisons. Statistical significance was defined as *p* < 0.05 for all analyses.

## 3 Results

### 3.1 TRIM52 Expression Was Upregulated Following DOX Treatment

**FIGURE 1:**
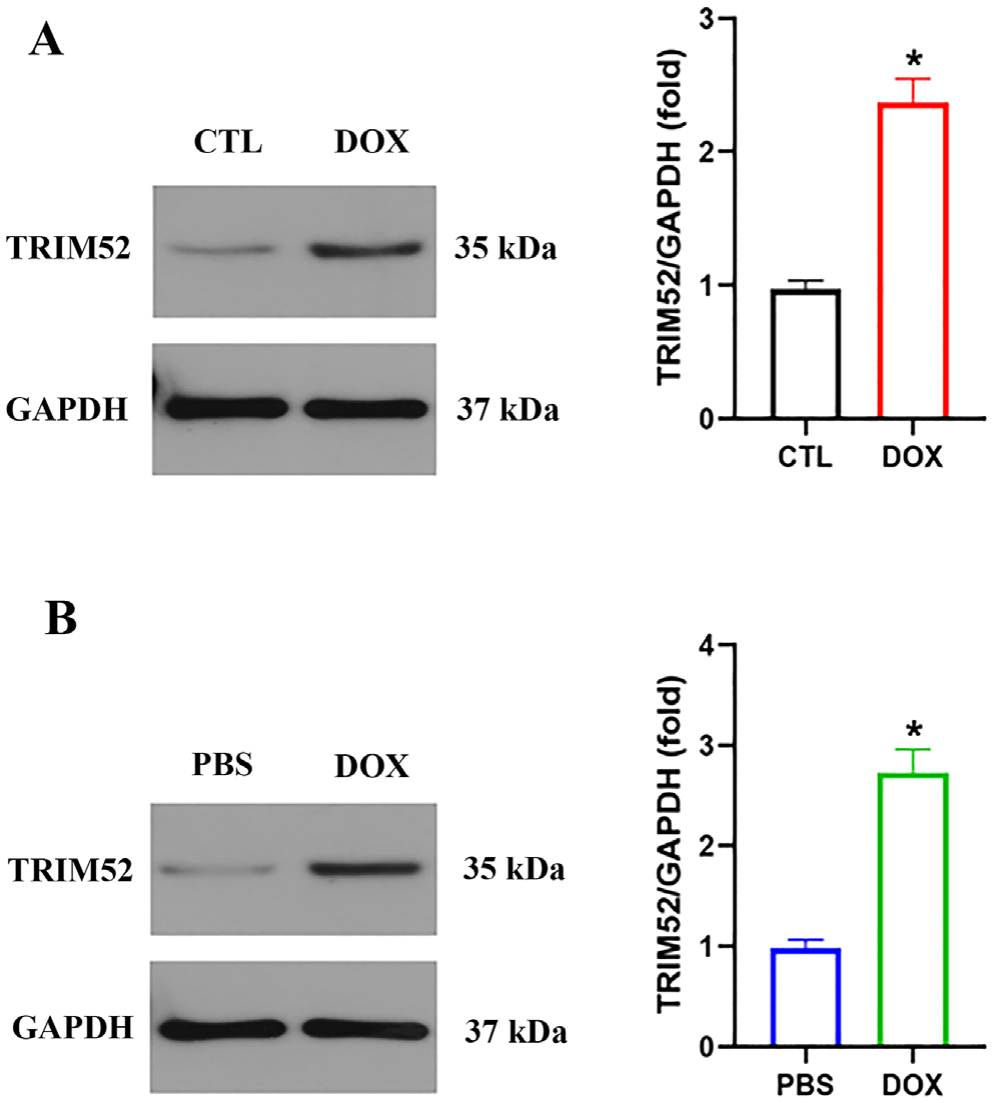
TRIM52 was elevated following DOX treatment. (A) Representative western blot and quantitative data of TRIM52 protein expression in mice (*n* = 3 per group). (B) Representative western blot and quantitative data of TRIM52 protein expression in neonatal mouse cardiomyocytes (*n* = 3 per group). *p < 0.05.

To investigate the role of TRIM52 in DIC, we first examined its expression patterns. Western blotting revealed that TRIM52 expression was significantly increased in ventricular tissues from DOX‐treated mice compared with controls (Figure 1A). Consistently, in vitro experiments showed a corresponding upregulation of TRIM52 protein in primary murine cardiomyocytes following DOX exposure (Figure 1B).

### 3.2 TRIM52 Deletion Attenuates DOX‐Induced Cardiac Injury

**FIGURE 2:**
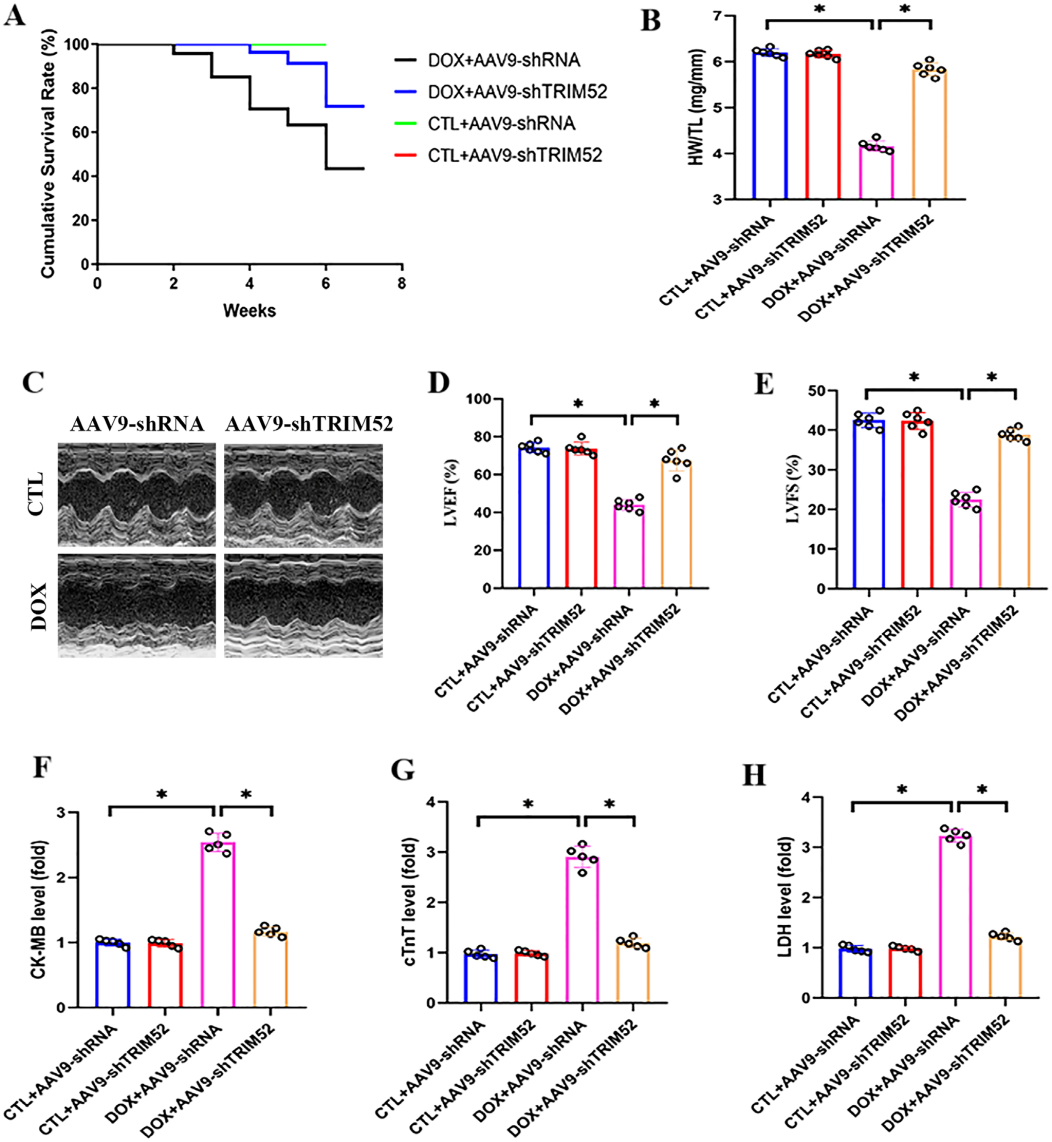
TRIM52 deficiency alleviated DOX‐induced cardiac injury. (A) Survival curve. (B) Statistical results of heart weight to tibia length (HW/TL) ratio (*n* = 6 per group). (C) Representative images of echocardiogram. (D and E) Statistical results of LVEF and LVFS (*n* = 6 per group). (F–H) Statistical results of serum levels of CK‐MB, cTnT, and LDH (*n* = 5 per group). *p < 0.05.

To investigate the role of TRIM52 in DIC, we delivered AAV9 vectors encoding TRIM52‐targeting constructs to silence TRIM52 in mice. DOX‐treated mice showed significantly reduced survival compared with control animals (Figure 2A). Notably, TRIM52 knockdown substantially improved survival in DOX‐challenged mice (Figure 2A). DOX administration also caused systemic deterioration, as reflected by a decreased heart weight‐to‐tibia length (HW/TL) ratio (Figure 2B). Consistently, echocardiography revealed marked reductions in LVEF and LVFS in the DOX group relative to the CTL group (Figure 2C–E). TRIM52 deficiency significantly alleviated these DOX‐induced impairments in cardiac function (Figure 2C–E). In line with these findings, serum biomarkers of myocardial injury (LDH, CK‐MB, and cTnT) were significantly elevated after DOX treatment, whereas TRIM52 silencing markedly reduced their levels compared with the DOX + control vector group (Figure 2F–H).

### 3.3 TRIM52 Deficiency Alleviates DOX‐Induced Cardiac Oxidative Stress

**FIGURE 3:**
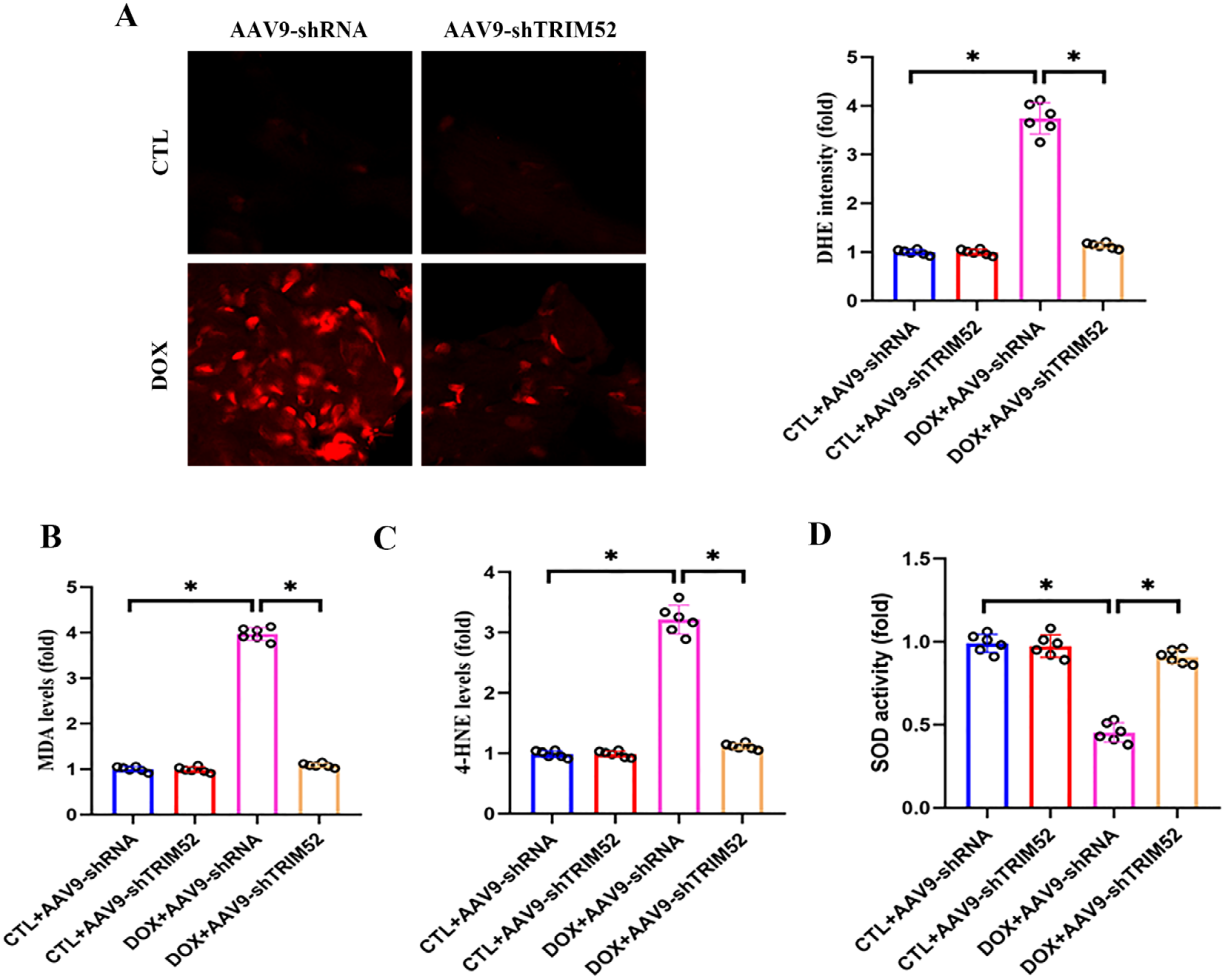
TRIM52 deficiency attenuated DOX‐induced oxidative stress. (A) Representative images and quantitative results of DHE stained heart sections (*n* = 5 per group). (B–D) Quantitative results of MDA, 4‐HNE and SOD activity in heart tissues (*n* = 5 per group). *p < 0.05. Bar = 50 μm.

Oxidative stress is a major pathogenic driver of DIC, yet the role of TRIM52 in redox regulation remains unclear. To determine whether TRIM52 contributes to DOX‐induced cardiac oxidative injury, we performed a systematic assessment of oxidative stress markers. DHE fluorescence analysis showed that cardiac ROS levels were markedly increased in DOX‐exposed mice compared with controls, whereas TRIM52 deficiency significantly suppressed ROS generation (Figure 3A). Consistently, biochemical assays further supported the antioxidative effects of TRIM52 depletion: TRIM52‐silenced mice exhibited substantially reduced lipid peroxidation markers (MDA and 4‐HNE) and enhanced SOD activity relative to DOX‐treated counterparts (Figure 3B–D). Moreover, the in vitro experimental results indicated that DOX markedly increased the number of TUNEL‐positive nuclei in NMCMs (Figure S2A). Importantly, TRIM52 silencing significantly reduced DOX‐induced apoptosis, aligning with our in vivo findings that TRIM52 deficiency alleviates DOX‐triggered myocardial injury. Consistent with the findings from the in vivo experiment, TRIM52 silencing also significantly alleviated DOX‐induced cardiomyocyte oxidative stress (Figure S2B–D).

### 3.4 TRIM52 Deletion Inhibits DOX‐Induced Cardiac Inflammatory Response

**FIGURE 4:**
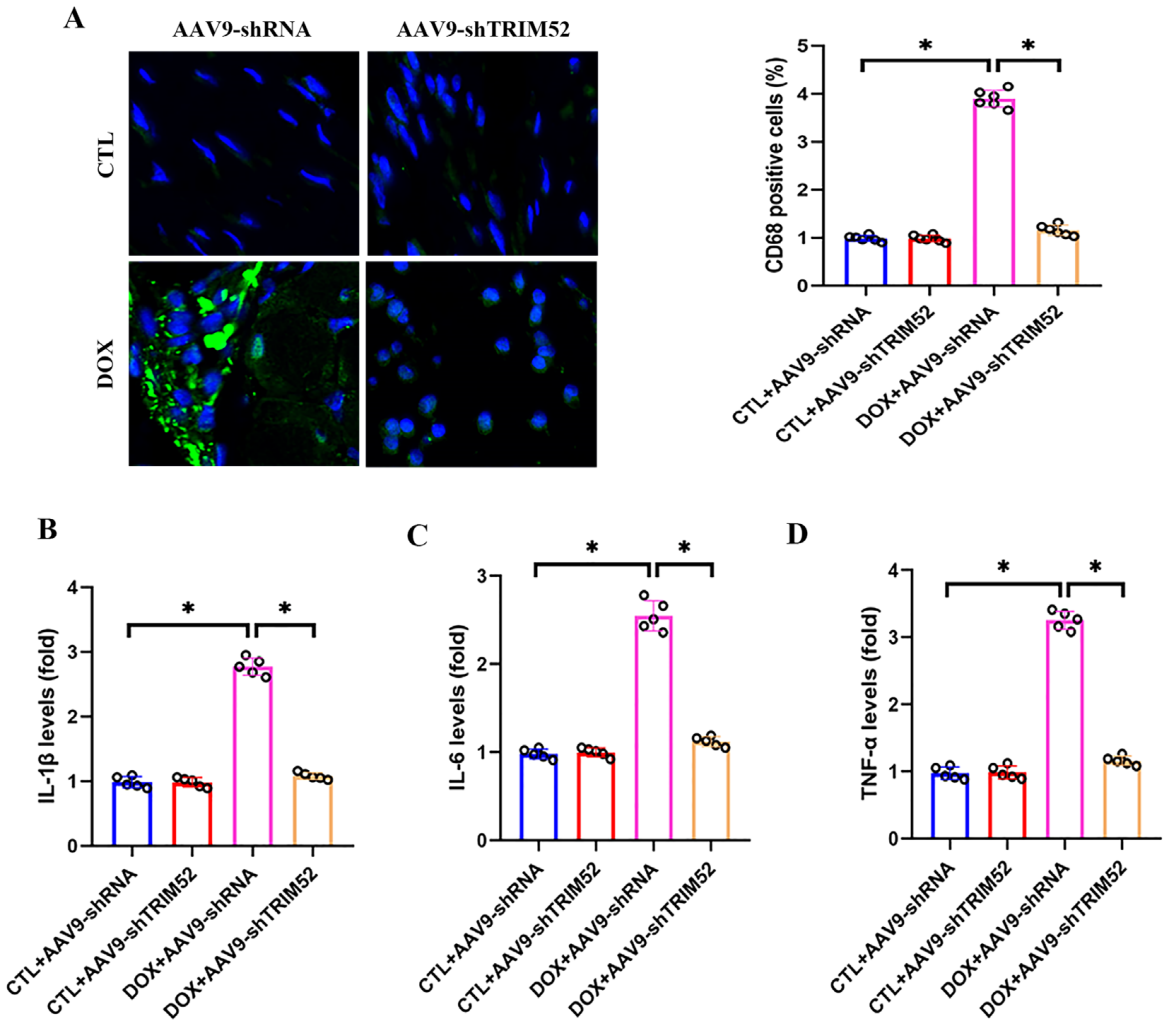
TRIM52 deficiency inhibited DOX‐induced inflammation. (A) Representative images and quantitative results of CD68‐stained heart sections (*n* = 5 per group). (B–D) Quantitative results of the serum levels of IL‐1β, IL‐6 and TNF‐α (*n* = 5 per group). *p < 0.05. Bar = 50 μm.

Inflammation is a central pathological mechanism in the progression of DIC. Immunofluorescence analysis revealed a marked increase in F4/80‐positive macrophage infiltration in the myocardium of DOX‐treated mice compared with controls, whereas TRIM52 knockdown substantially attenuated this response (Figure 4A). Consistently, ELISA‐based cytokine profiling showed that circulating IL‐6, IL‐1β, and TNF‐α levels were significantly elevated after DOX exposure and were largely normalised by TRIM52 silencing (Figure 4B–D).

### 3.5 PI3K/Akt Signalling Axis Contributes to the Protective Effect of TRIM52 Ablation Against DIC

**FIGURE 5:**
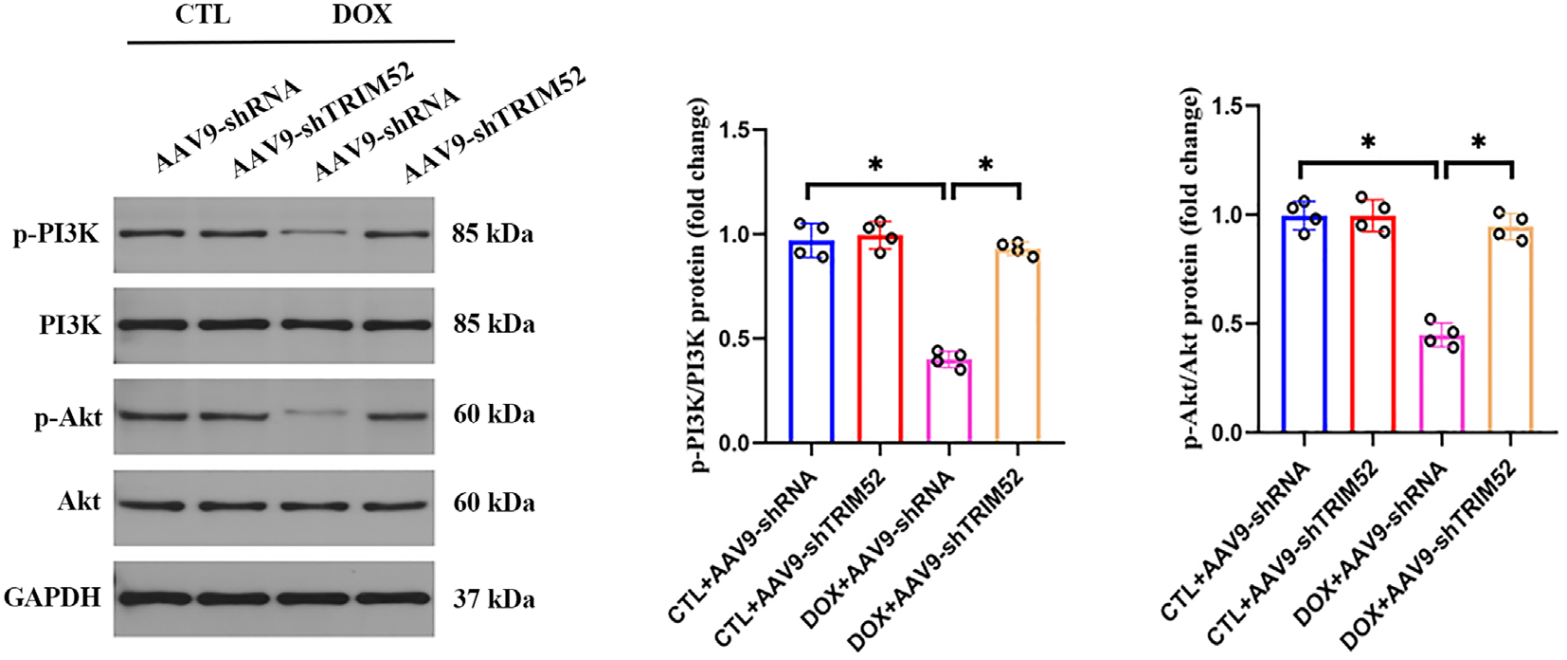
PI3K/Akt signalling contributes to the cardioprotective effect TRIM52 deficiency. Representative western blot and quantitative data of p‐PI3K, PI3K and p‐Akt, Akt protein expression (*n* = 4 per group). *p < 0.05.

The PI3K/AKT signalling axis is an important regulator of DIC pathogenesis; however, whether it mediates the cardioprotective effects of TRIM52 ablation remains unclear. Phosphoprotein analyses showed that DOX challenge significantly suppressed PI3K/AKT activity, as evidenced by reduced phosphorylation of PI3K and AKT compared with controls. Notably, TRIM52 ablation restored PI3K/AKT signalling, with marked increases in p‐PI3K and p‐AKT levels. These findings suggest that reactivation of PI3K/AKT may underlie, at least in part, the protective effects of TRIM52 loss in DIC (Figure 5).

### 3.6 Pharmacological Blockade of PI3K/Akt Signalling Negated the Cardioprotective Benefits Conferred by TRIM52 Ablation in the Context of DOX‐Induced Myocardial Injury

**FIGURE 6:**
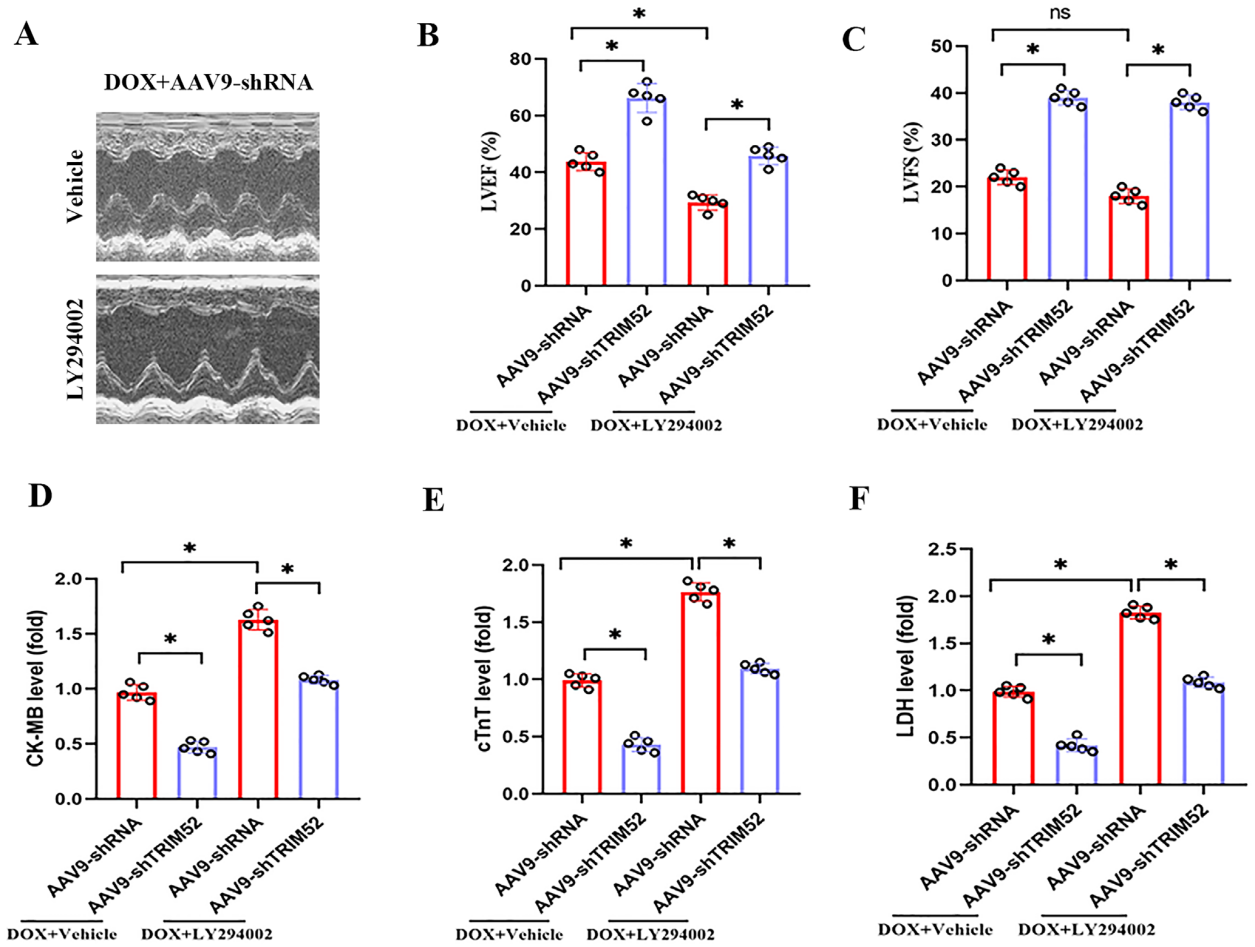
Pharmacological blockade of PI3K/Akt signalling negated the cardioprotective benefits conferred by TRIM52 ablation in the context of DOX‐induced myocardial injury. (A) Representative images of echocardiogram. (B and C) Statistical results of LVEF and LVFS (*n* = 5 per group). (D–F) Statistical results of serum levels of CK‐MB, cTnT and LDH (*n* = 5 per group). *p < 0.05.

Pharmacological validation was performed to confirm the involvement of PI3K/AKT signalling in TRIM52‐mediated cardioprotection. Treatment with LY294002, a PI3K inhibitor, abolished the preservation of cardiac function (LVEF and LVFS) observed in TRIM52‐deficient mice following DOX challenge, as shown by echocardiographic analyses (Figure 6A–C). Consistently, serum biomarkers of myocardial injury (CK‐MB, cTnT, and LDH) were significantly increased in LY294002‐treated groups, indicating a reversal of the cardioprotective effects of TRIM52 deficiency against DOX‐induced cardiac damage (Figure 6D–F).

### 3.7 Pharmacological Suppression of PI3K/Akt Signalling Abrogated the Antioxidative and Anti‐Inflammatory Protection Conferred by TRIM52 Ablation in DOX‐Challenged Models

**FIGURE 7:**
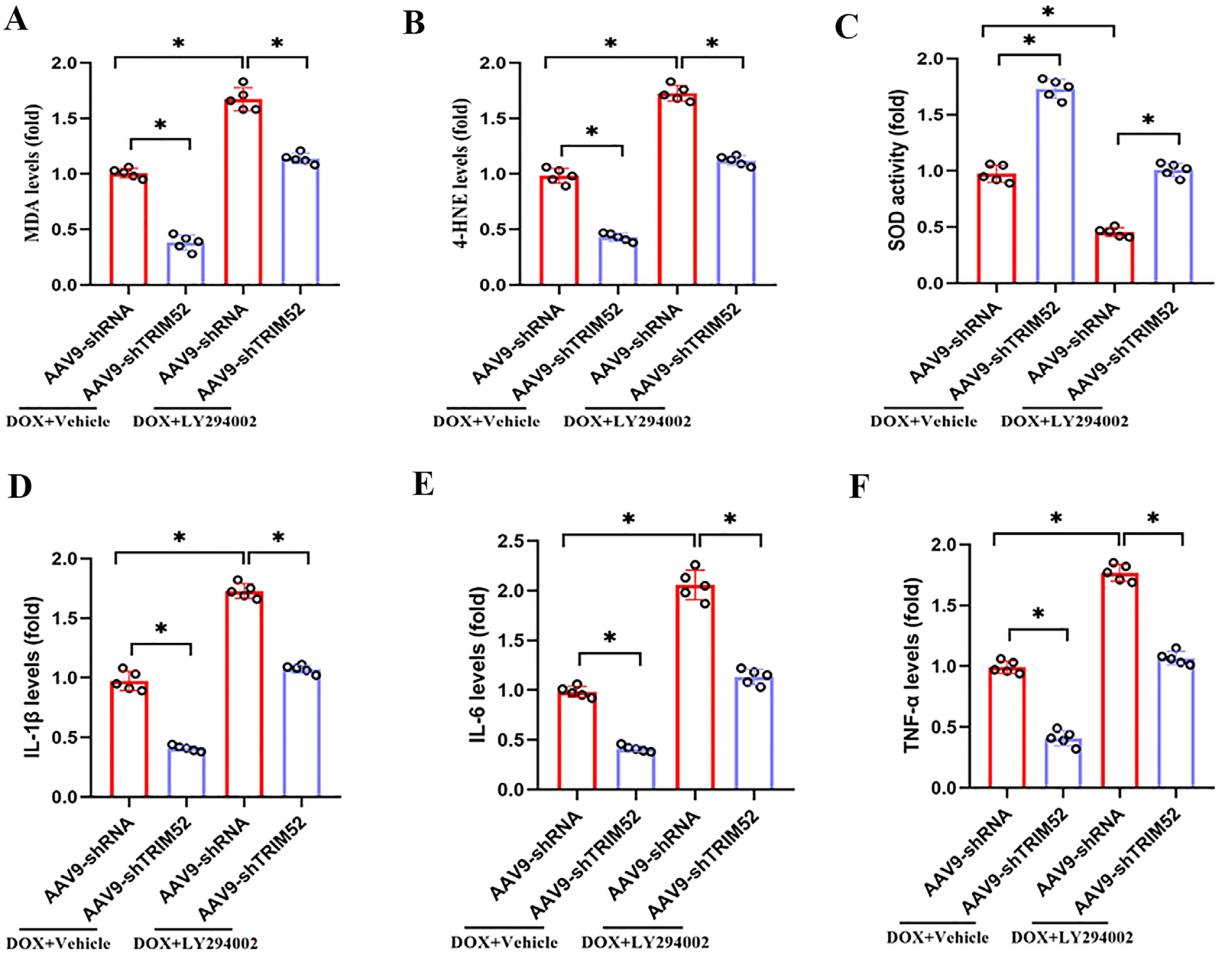
Pharmacological suppression of PI3K/Akt signalling abrogated the antioxidative and anti‐inflammatory protection conferred by TRIM52 ablation in DOX‐challenged models. (A–C) Quantitative results of MDA, 4‐HNE levels and SOD activity in different groups (*n* = 5 per group). (D–F) Quantitative analysis of levels of IL‐1β, IL‐6 and TNF‐α (*n* = 5 per group). *p < 0.05.

Additionally, we assessed whether inhibition of PI3K/AKT signalling influences DOX‐induced oxidative stress and inflammatory responses. LY294002 administration markedly aggravated DOX‐triggered cardiac oxidative stress, as evidenced by increased MDA and 4‐HNE levels and reduced SOD activity, and it reversed the antioxidant benefits conferred by TRIM52 deletion after DOX exposure (Figure 7A–C). Moreover, LY294002 significantly intensified DOX‐induced inflammation, reflected by elevated IL‐6, IL‐1β, and TNF‐α levels, and similarly abrogated the anti‐inflammatory effects associated with TRIM52 deficiency in the setting of DOX treatment (Figure 7D–F).

## 4 Discussion

This study identifies TRIM52 as a key mediator of DIC. We found that TRIM52 is upregulated in DOX‐stressed hearts and cardiomyocytes, and that genetic deletion of TRIM52 mitigates cardiac dysfunction and structural injury while reducing oxidative stress and inflammatory cytokine release. Mechanistically, these protective effects are closely linked to preservation of PI3K/AKT signalling, as pharmacologic inhibition largely abolishes the benefits of TRIM52 deficiency. Collectively, our data suggest that targeting TRIM52 may represent a promising strategy to prevent or treat anthracycline‐related myocardial injury.

Members of the TRIM protein family are increasingly recognised as critical regulators of cardiovascular pathology. For example, TRIM27 is markedly downregulated in murine hearts subjected to myocardial ischemia/reperfusion (I/R) injury and in cardiomyocytes exposed to hypoxia/reoxygenation (H/R); cardiomyocyte‐specific TRIM27 knockout further enlarges infarct size and worsens cardiac dysfunction in I/R mice [16]. Similarly, Trim72 expression declines in failing human hearts and in aged murine hearts, and restoring Trim72 improves cardiac performance in older animals [17]. In the present study, we show for the first time that cardiac TRIM52 is upregulated in response to DOX challenge. Comparable alterations in other TRIM proteins under DOX stress have also been reported: DOX increases myocardial TRIM35 expression [18] and significantly elevates Trim72 levels in H9c2 cells [19]. Consistent with these observations, our data indicate that DOX robustly induces TRIM52, likely as part of the myocardial stress response. Importantly, functional experiments demonstrated that TRIM52 deficiency markedly alleviates DOX‐induced cardiac dysfunction and structural injury, suggesting that TRIM52 acts as a maladaptive regulator in this context. Collectively, our findings not only document DOX‐triggered TRIM52 induction but also establish its pathological relevance, supporting the concept that elevated TRIM52 contributes to DIC and may represent a therapeutically actionable target.

Oxidative stress is a central mediator of DIC. In cardiomyocytes, DOX provokes excessive production of ROS, driving lipid peroxidation, depleting antioxidant defences, and damaging cellular organelles [20]. TRIM proteins are increasingly recognised as modulators of myocardial redox homeostasis: TRIM72 preserves mitochondrial integrity during I/R‐induced oxidative stress [21], whereas TRIM8 knockdown limits ROS accumulation and enhances superoxide dismutase and glutathione peroxidase activity in H9c2 cells subjected to H/R [13]. Our data extend this concept by identifying TRIM52 as a key regulator of cardiac redox balance. Under DOX challenge, TRIM52 deletion markedly reduced myocardial ROS levels and oxidative damage markers while restoring endogenous antioxidant capacity, as reflected by increased superoxide dismutase activity. In contrast, wild‐type mice with pronounced DOX‐induced TRIM52 upregulation developed a more severe redox imbalance. Collectively, these findings indicate that TRIM52 amplifies DOX‐induced oxidative injury in the heart.

Beyond oxidative stress, DOX‐induced myocardial injury is characterised by a vigorous sterile inflammatory response. DOX prompts cardiomyocytes to release damage‐associated molecular patterns (DAMPs) that engage innate immune sensors such as Toll‐like receptor 4 (TLR4). This activation triggers downstream pathways—most notably NF‐κB—resulting in robust production of pro‐inflammatory cytokines [22, 23]. TRIM family proteins have emerged as key regulators of cardiac inflammation. For example, TRIM27 attenuates myocardial I/R injury by restraining inflammatory signalling [16]; TRIM72 suppresses NF‐κB activation to alleviate age‐related heart failure [17]; and TRIM37 exerts similar effects by ubiquitinating NF‐κB‐associated proteins [24]. Consistent with this paradigm, we found that TRIM52 deletion markedly reduces the inflammatory burden in DOX‐treated hearts, as evidenced by lower expression of pro‐inflammatory cytokines (e.g., TNF‐α and IL‐6) and diminished leukocyte infiltration. These findings indicate that TRIM52 facilitates the DOX‐triggered inflammatory cascade. Prior work supports this conclusion: in IL‐1β‐stimulated in vitro models, elevated TRIM52 amplifies cytokine production by enhancing the TLR4/NF‐κB axis, whereas TRIM52 silencing attenuates TLR4 and p65 NF‐κB activation and thereby blunts inflammation [25]. Collectively, our results identify TRIM52 as a pro‐inflammatory mediator that aggravates DOX‐induced myocardial damage and suggest that targeting TRIM52 may provide anti‐inflammatory cardioprotection.

The PI3K/AKT pathway is pivotal for cardiomyocyte survival and stress resistance. Activated AKT promotes cell viability, inhibits apoptosis, and—via downstream effectors such as Nrf2—strengthens both antioxidant and anti‐inflammatory defences [26]. Numerous studies have shown that DOX suppresses PI3K/AKT activation, whereas re‐engaging this pathway mitigates DOX‐induced oxidative stress and inflammation [8, 9]. Our data reveal that TRIM52 modulates these effects through PI3K/AKT signalling. Under DOX challenge, TRIM52 knockout mice exhibited significantly higher myocardial AKT phosphorylation than wild‐type controls, indicating more robust PI3K/AKT activity. Importantly, pharmacological inhibition of this pathway abolished these differences and nullified the cardioprotective benefits of TRIM52 loss. These findings suggest that the antioxidant and anti‐inflammatory protection conferred by TRIM52 deletion is largely dependent on PI3K/AKT signalling. Recent studies further link TRIM proteins to this cascade; for example, TRIM8 knockdown protects H9c2 cells from H/R injury by activating PI3K/AKT [13]. To our knowledge, our work is the first to establish a comparable connection for TRIM52, providing mechanistic insight into its pathogenic role and a rationale for targeting PI3K/AKT‐related pathways in DOX cardiotoxicity.

### 4.1 Limitations

This study has several limitations. First, we relied on male C57BL/6 TRIM52‐deficient mice and neonatal murine cardiomyocytes, which may limit direct extrapolation to human pathophysiology. Validation of TRIM52 expression in human cardiac samples and functional studies in human iPSC‐derived cardiomyocytes would strengthen clinical relevance. Second, although we linked PI3K/AKT signalling to TRIM52‐regulated oxidative and inflammatory responses, the precise proximal molecular targets of TRIM52 remain undefined. Future studies using ubiquitin‐proteomics, phosphoproteomics, and targeted interaction assays will be necessary to identify direct substrates or binding partners within this axis. Third, while genetic ablation is mechanistically informative, it does not fully reflect translational feasibility. The development of TRIM52‐targeted therapies—such as cardiac‐selective RNA‐based approaches or small‐molecule modulators—will require rigorous evaluation of tissue specificity, delivery efficiency, and off‐target effects in preclinical models.

## 5 Conclusions

TRIM52 deletion activates the PI3K/AKT signalling pathway, mitigating DOX‐induced oxidative and inflammatory myocardial damage. These results identify TRIM52 as a potential therapeutic target for the prevention of DIC.

## Author Contributions

W.D. and J.S. designed experiments; Z.Z. and H.C. collected data; Y.H., J.Z. and T.D. analysed experimental results. Z.Z. wrote the manuscript; J.S., W.D. approved the manuscript. All authors read and approved the final manuscript.

## Funding

The study was supported by Zhejiang Provincial Health and Wellness Industry Science and Technology Plan (2025HY0968, 2025HY0940), Zhejiang Provincial Natural Science Foundation of China (MRY26H170012, LTGY24H020001), Zhejiang Vanguard and Leading Goose R&D Program (2026C02A1259) (SD2), the Project of Science and Technology on Traditional Chinese Medicine in Zhejiang Province (2023ZR131, 2023ZL158), the Key Public Welfare Project of Ningbo (2024S030), the Key Technology R&D Program of Ningbo (2022Z149).

## Ethics Statement

The institutional ethics committee of the First Affiliated Hospital of Ningbo University has approved this study.

## Consent

All authors have consented to the submission of the article to the journal.

## Conflicts of Interest

The authors declare no conflicts of interest.

## Supporting information


**Figure S1:** The efficiency of TRIM52 knockdown by AAV9‐shTRIM52.

**Figure S2:** (A) Representative images and quantitative results of TUNEL‐positive cells in different groups (*n* = 5 per group). (B–D) Quantitative results of MDA, 4‐HNE and SOD activity in different groups (*n* = 5 per group). *p < 0.05. Bar = 50 μm.

## Data Availability

The data that support the findings of this study are available on request from the corresponding author. The data are not publicly available due to privacy or ethical restrictions.
