# Supplementary figures and images for: TRIM52 Protects Against Doxorubicin‐Induced Cardiac Inflammation, Oxidative Stress and Cardiac Injury
Source: J Cell Mol Med. 2026 Jan 9;30(1):e71016. doi: 10.1111/jcmm.71016 (PMC12784278; doi:10.1111/jcmm.71016)

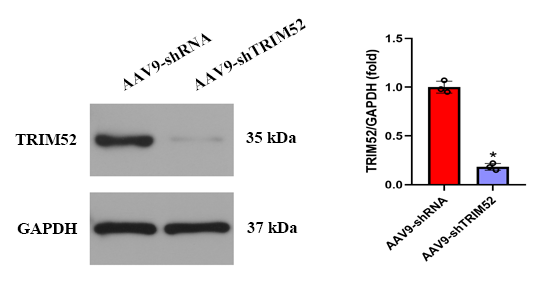

Supplement: Supplementary file 1 — Figure S1: The efficiency of TRIM52 knockdown by AAV9‐shTRIM52. [file JCMM-30-e71016-s001.tif]

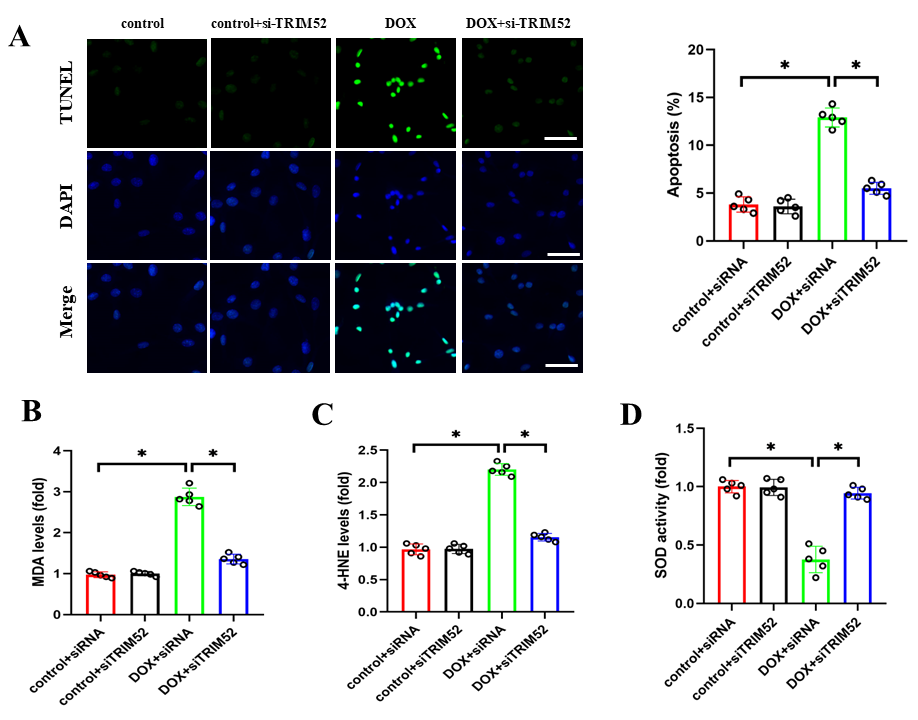

Supplement: Supplementary file 2 — Figure S2: (A) Representative images and quantitative results of TUNEL‐positive cells in different groups (n = 5 per group). (B–D) Quantitative results of MDA, 4‐HNE and SOD activity in different groups (n = 5 per group). *p < 0.05. Bar = 50 μm. [file JCMM-30-e71016-s002.tif]
